# Predictive value of aberrant right subclavian artery for fetal chromosome aneuploidy in women of advanced maternal age

**DOI:** 10.1186/s12884-021-03626-7

**Published:** 2021-02-18

**Authors:** Li-Ping Chen, Yong-Feng Lai, Xiao-Hong Zhong, Jian-Hong You, Jiang-Hua Chen, Jing-Xian Xie, Xiao-Kang Chen, Xiao-Yan Chen, Guo-Rong Lyu

**Affiliations:** 1 Department of Ultrasound, Women and Children’s Hospital, School of Medicine, Xiamen University, NO. 10 Zhenhai Road, Siming District, Xiamen, 361000 Fujian People’s Republic of China; 2 Department of Ultrasound, Wuping County Hospital, Longyan, 364300 Fujian People’s Republic of China; 3 Department of Ultrasound, Zhongshan Hospital of Xiamen University, Xiamen, 361000 Fujian People’s Republic of China; 4 Department of Obstetrics and Gynecology, Women and Children’s Hospital, School of Medicine, Xiamen University, Xiamen, 361000 Fujian People’s Republic of China; 5 Department of Ultrasound, Children’s Hospital of Fudan University Xiamen Branch, Xiamen Children’s Hospital, Xiamen, 361000 Fujian People’s Republic of China; 6 Department of Clinical Medicine, Quanzhou Medical College, Quanzhou, 362000 Fujian People’s Republic of China

**Keywords:** Ultrasonography, Fetus, Aberrant right subclavian artery, Chromosomal abnormalities, Advanced maternal age

## Abstract

**Background:**

In the entire population, an aberrant right subclavian artery (ARSA) is closely associated with chromosomal abnormalities. ARSA with additional ultrasonic findings would increase risk of chromosomal abnormalities. The risk of fetal chromosomal abnormalities increased exponentially with the maternal age. These risks in the advanced maternal age (AMA) group are uncertain. This study aimed to determine the incidence of ARSA in Chinese AMA and non-AMA women and the frequency of aneuploidy among AMA and non-AMA women with ARSA.

**Methods:**

This retrospective study included 13,690 singleton pregnancies, were divided into AMA and non-AMA groups. Integrated obstetric ultrasonic screening, biochemical screening, noninvasive prenatal screening, and fetal karyotype analysis were analyzed.

**Results:**

The overall incidence of ARSA was 0.69%, with no difference between age groups. The incidence of chromosomal abnormalities in the AMA group (37 / 2860) was much higher than that of the non-AMA group. The risk of chromosomal abnormalities significantly increased with both ARSA detected and additional ultrasound findings. With combined ARSA and AMA, the likelihood of the incidence of chromosomal abnormalities increased. Chimerism (45X / 46XX) was found with isolated ARSA in AMA pregnancies.

**Conclusion:**

There is a high prevalence of chromosomal abnormalities in fetuses of AMA women. ARSA increases the risk of chromosomal abnormalities in both age groups, especially combined with ARSA. When ARSA occurs in AMA women, it confers a high likelihood of chromosomal abnormalities.

## Background

Advanced maternal age (AMA) is defined as conception and delivery at 35 years or older [1–4]. According to the Office for National Statistics, in 2013, 20% of births in England and Wales were to women aged 35 years or over; mothers’ average age has dramatically increased over time [1]. In China, the proportion of AMA women increased from 10.1% in 2011 to 20.5% in 2016, and it increased further after the institution of the “two-child policy” [2].

Maternal age closely associates with pregnancy complications such as preeclampsia, stillbirth, and fetal anomalies [1–3]. The risk of fetal chromosomal abnormalities increased with the maternal age exponentially. For example, Down’s syndrome’s overall incidence is one in every 800 births, while it climbs to 1.44 in every 100 births in AMA women [4].

Chen et al. stated that there is no need for AMA women to directly undergo invasive prenatal diagnosis [5]. Nevertheless, there remains the possibility of other associated fetal structural abnormalities in AMA women, including aberrant right subclavian artery (ARSA).

ARSA is often detected by trained ultrasound operators during prenatal ultrasonography regardless of pregnancy trimester [6]. The incidence of ARSA as an isolated abnormality in healthy populations is at about 1 to 2% [7]. Chaoui et al. reported the prevalence of ARSA in fetuses with Down’s syndrome for the first time and suggested that ARSA could be a new soft marker for trisomy 21 [8, 9]. The prevalence of ARSA was 1.02% in euploid fetuses and 23.6% in Down’s syndrome fetuses [10]. Therefore, ARSA appears to be a reasonably reliable ultrasound clue for fetal chromosomal abnormalities, especially congenital cardiac defects and aneuploid abnormality [6–10].

Most studies found that isolated ARSA had no clinical significance and did not serve as an invasive prenatal chromosomal test [8–12]. An invasive procedure was offered to all patients with intermediate-risk and retrotracheal ARSA [10]. Fehmi et al. suggested fetuses with ARSA and aneuploidy relevant soft ultrasonic features, AMA, and abnormal biochemical screening should undergo amniocentesis [12]. They concluded that in fetuses with ARSA, karyotyping could be offered to detect Down’s syndrome if any risk factors were present.

However, in most studies, the predictive value of ARSA in AMA and non-AMA women has not been compared. In AMA women, it is unclear whether ARSA is a useful predictor for fetal chromosomal abnormalities or whether it is necessary for them to undergo invasive screening. Hence, this study’s purposes were: 1) to determine the incidence of both isolated and non-isolated ARSA in AMA and non-AMA women in southern Fujian of China, and 2) to assess the association between fetal chromosomal abnormalities and ARSA with or without additional ultrasound findings in AMA pregnancy.

## Methods

### Study population and device

The retrospective study of 13,690 single pregnancies with complete data was performed at Zhongshan Hospital of Xiamen University and Women and Children's Hospital, School of Medicine, Xiamen University, from September 2015 to January 2018. AMA was defined as conception and delivery at 35 years of age or older [1]. The women were classified into two age categories based on their due dates: those younger than 35 (the non-AMA group) and those equal to or older than 35 (the AMA group). The Medical Ethics Committee of Xiamen University approved the study, and written informed consent was obtained from the participants. Prenatal ultrasonography screening was performed using a transabdominal high-resolution probe (C4–8-D probe and 9 L-D high-frequency probe; Voluson E8/E10; GE Medical Systems, Zipf, Austria).

### Study design

**Fig. 1:**
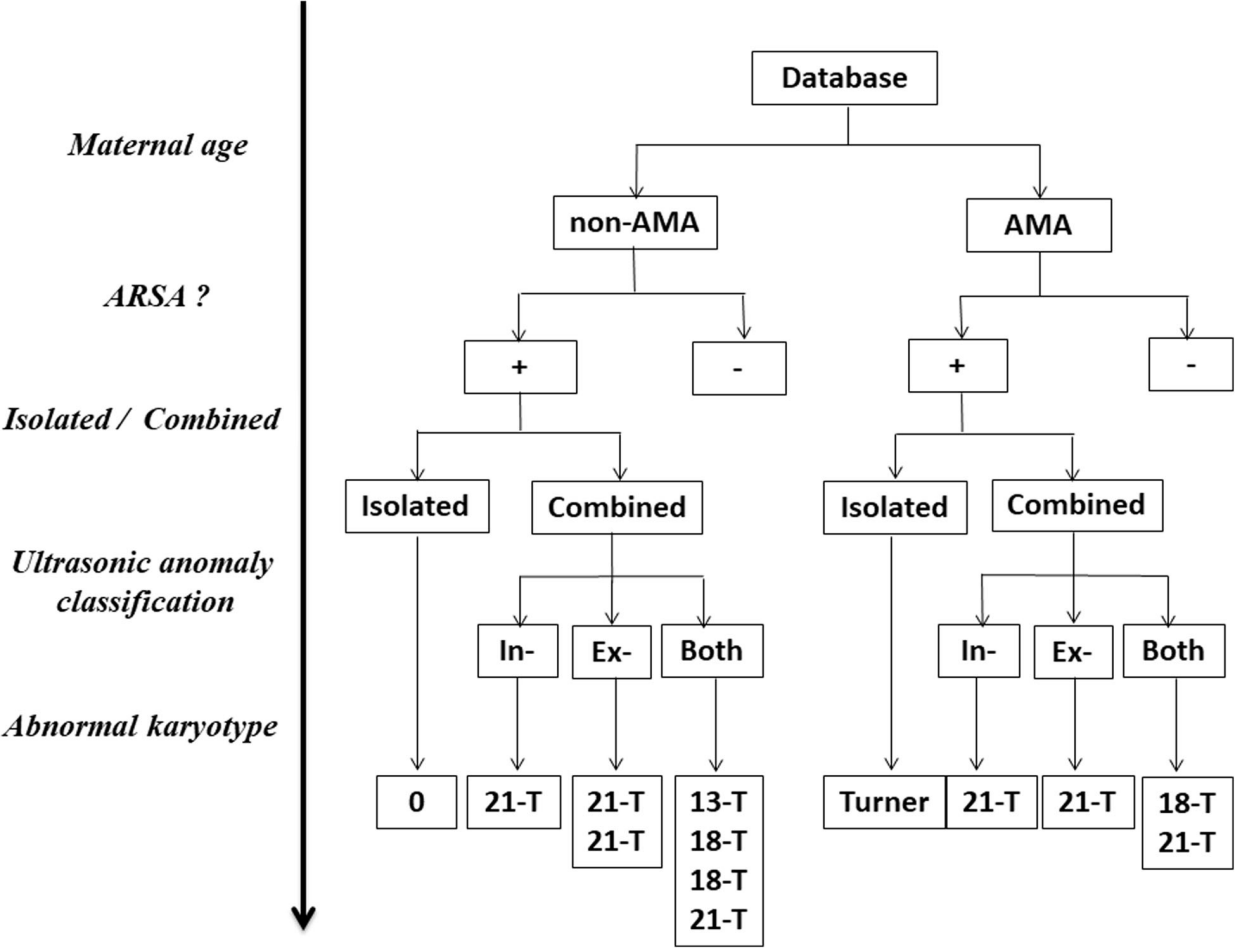
The flowchart of our study. AMA, advanced maternal age; non-AMA, appropriate maternal age; ARSA; aberrant right subclavian artery; In-, Intracardiac malformation; Ex-, Extracardiac malformation; Both, Intracardiac and extracardiac malformation

The information of all included pregnant women was retrieved from our computerized database. The complete data was including fetal ultrasonic prenatal screening (grades I - III and fetal echocardiography), biochemical screening, noninvasive prenatal testing (NIPT), and fetal karyotype analysis. All participants (whether ARSA or not) underwent routine ARSA screening and were followed up until birth by neonatal echocardiography. All ARSA fetuses and suspected cases were diagnosed by two physicians (with prenatal diagnosis qualification and rich experience) and confirmed by at least one postnatal follow-up review. The relevant prenatal diagnostic information was classified and summarized, including ultrasonography abnormities, soft markers, serum screening, non-invasive and invasive karyotype analysis, and chromosomal microarray. Prenatal consultation with noninvasive or invasive karyotype analysis was recommended for all fetuses with ARSA. In pregnant women who did not undergo invasive karyotype analysis, noninvasive DNA testing, detailed prenatal testing, and neonatal follow-up revealed no significant aneuploidy or karyotype abnormalities that were considered normal. All aborted fetuses underwent autopsies with informed consent. The flow chart of this study is shown in Fig. 1.

### Method of ARSA detection

**Fig. 2:**
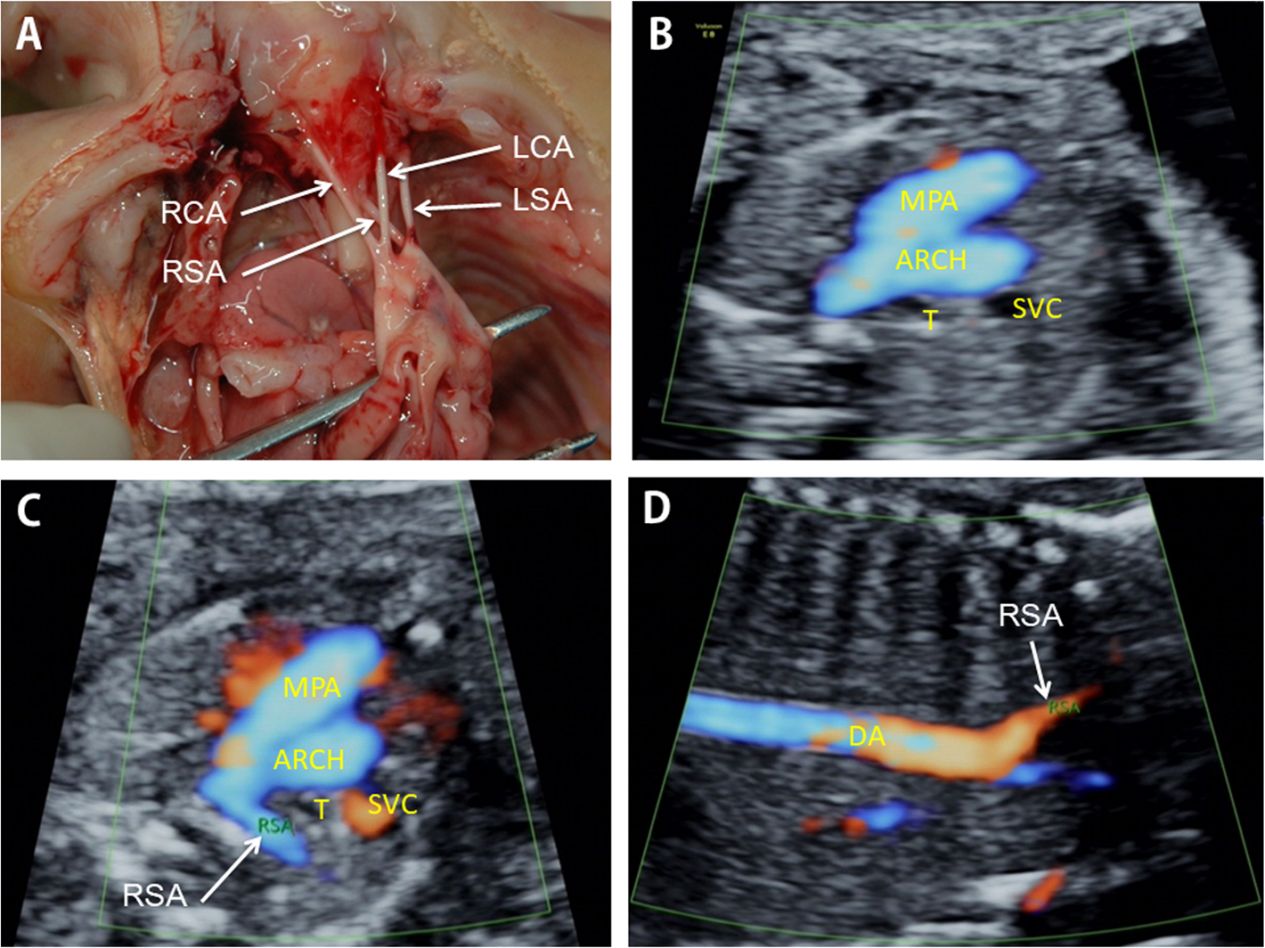
Inspection of fetal aberrant right subclavian artery. **a**: Anatomical diagram of fetal ARSA. **b**: The three vessels and trachea view of normal fetuses. **c**: Fetal ARSA in the three vessels and trachea view. **d**: Fetal ARSA in the coronal view. ARCH, aorta; DA, descending aorta; LCA, left carotid artery; LSA, left subclavian artery; MPA, main pulmonary artery; RCA, right carotid artery; RSA, right subclavian artery; SVC, superior vena cava; T, trachea

After routine examination, the fetal heart mode was turned on, and the local amplification function was adjusted to display the section. The angle of ARSA and incident sound wave was ensured less than 30. Axial view (the three-vessel and trachea view), longitudinal view, and coronal view were conventionally observed to screen for ARSA [13]. When ARSA was found in two-dimension mode, the Doppler velocity was adjusted to 15 cm/s - 30 cm/s to verify the diagnosis. ARSA departed from the descending aorta’s origin, namely the junction of the aortic arch and ductal arch, traveled between the trachea and the vertebra, and extended toward the right shoulder. Anatomical and ultrasonic diagrams of fetal ARSA are shown in Fig. 2.

### Statistical analysis

Statistical analysis was performed using SPSS software, version 20.0 (IBM Corporation, Somers, NY, USA). The chi-square and Fisher’s exact tests were utilized to compare chromosomal abnormalities and ARSA incidences between groups. The Mantel-Haenszel test was used to investigate possible effect modification of age on the association between ARSA and chromosomal abnormalities. The odds ratio (OR) was calculated, and the Breslow-Day-Tarone test was used to test homogeneity. When contingency tables with structural zero, the homogeneity was tested using logarithmic-transformation-based statistics. Likelihood ratios with 95% confidence intervals (CI) were calculated. *P* < 0.05 was considered statistically significant.

## Results

### Demographic data and general characteristics

During this period, a total of 13,690 singleton pregnancies (ranging from 16 weeks + 0 days to 38 weeks + 5 days) were recruited, including 10,830 non-AMA women with an average age of 27.1 ± 4.15 years and 2860 AMA women with an average age of 38.3 ± 3.48 years. Among these, ARSA was prenatally detected with an overall incidence of 0.69% (95 / 13,690), including 63 cases (63 / 95, 66.32%) with isolated ARSA. In the non-AMA group, 70 of 10,830 fetuses were prenatally diagnosed with an ARSA, for an incidence of 0.65%. In the AMA group, 25 of 2860 fetuses had an ARSA, with a 0.87% incidence. Chromosomal abnormalities were detected with a rate of 0.75% (102 / 13,690), including 0.63% (65 / 10,380) in non-AMA group and 1.29% (37 / 2860) in AMA group. Demographic data and general characteristics are displayed in Table 1.

**Table 1:** Demographic data and general characteristics in our study

	AMA	non-AMA	Total
Case	2860	10,830	13,690
Maternal age (y)	38.3 ± 3.48	27.1 ± 4.15	32.1 ± 4.71
Gestinatal age (w)	24 ± 2.11	26 ± 3.75	25 ± 3.43
Chromosomal abnormalities	1.29% (37/2860)	0.60% (65/10,830)	0.75% (102/13,690)
ARSA positive	0.87% (25/2860)	0.65% (70/10,830)	0.69% (95/13,690)
Isolated ARSA	60.00% (15/25)	68.57% (48/70)	66.32% (63/95)
Combined anomaly	35.13% (10/25)	31.43% (22/70)	33.68% (32/95)
Intracardiac malformation	12% (3/25)	7.14% (5/70)	8.42% (8/95)
Extracardiac malformation	12% (3/25)	11.43% (8/70)	11.58% (11/95)
Both	16% (4/25)	12.86% (9/70)	13.68% (13/95)
ARSA with Chromosomal abnormalities	20% (5/25)	10% (7/70)	12.63% (12/95)
ARSA negtive with Chromosomal abnormalities	1.13% (32/2835)	0.54% (58/10760)	0.66% (90/13595)

*ARSA* Aberrant right subclavian artery, *AMA* Advanced maternal age, *non-AMA* appropriate maternal age

Of the isolated ARSA fetuses, 63 (66.32%) were born, including 15 (60.00%) in the AMA group and 48 (68.57%) in the non-AMA group. Only one of the simple ARSA cases in the AMA group was detected with sex chromosome aneuploidies (single-nucleotide array arr (1–22) × 2,(X)× 1–2), and no obvious abnormality was found in the remainder during the clinical follow-up observation. The mother in question had no pregnancy risk factors or complications with critical risk on Down’s syndrome screening. Sex chromosome aneuploidies were first suggested by cell-free DNA detection and verified by amniocentesis karyotype analysis. This case of isolated ARSA might have been only a random event and was excluded in statistical analysis.

Of the 32 cases of ARSA with additional structural malformation, 28 ended in pregnancy termination, and 11 had abnormal karyotypes. There were four patients in the AMA group and seven in the non-AMA group, including trisomy 13, trisomy 18, and trisomy 21. The other four normal karyotype fetuses were delivered uneventfully, associated with a choroid plexus cyst, unilateral renal agenesis, a persistent left superior vena cava, and pulmonary sequestration.

### The incidence rate of chromosomal abnormalities

In the ARSA group, the incidence of chromosomal abnormalities was 20.00% (5 / 25) in the AMA group and 10.00% (7 / 70) in the non-AMA group. Following-up on fetuses with combined ARSA and chromosomal abnormalities, we found 58.33% (7 / 12) with trisomy 21, 25.00% (3 / 12) cases with trisomy 18, 8.33% (1 / 12) cases with trisomy 13, and 8.33% (1 / 12) cases with chimeric Turner syndrome (45X / 46XX). Among these combined ARSA, the incidence of chromosomal abnormalities was 34.38% (11 / 32), and the incidence of trisomy 21 was 21.88% (7 / 32), including 30.00% (3 / 10) in the AMA group and 18.18% (4 / 22) in the non-AMA group. More information is displayed in Table 2.

**Table 2:** Follow-up on fetus with combined ARSA and chromosomal abnormalities

Numerical order	Age cohorts	Pregnant week	Additional anomalies (intracardiac or extracardiac)	Serum screening result	Cell-free DNA	Karyotype	Outcome
NO.1	AMA	32	perimembranous ventricular septal defect; single umbilical artery; pyelectasis	positive	high risk	21-T	terminated
NO.2	AMA	26	unilateral cleft lip with alveolar cleft; single renal cyst	negative	high risk	21-T	terminated
NO.3	AMA	23	tetralogy of Fallot;	negative	high risk	21-T	terminated
NO.4	AMA	22	strawberry head; unilateral strephenopodia; fingers flexion and overlap; left ventricular dysplasia	positive	high risk	18-T	terminated
NO.5	non-AMA	23	mild lateral ventricular dilatation; slight tricuspid regurgitation; eyes spacing widened	positive	high risk	21-T	terminated
NO.6	non-AMA	32	omphalocele; angle of iliac ala increases	negative	high risk	21-T	terminated
NO.7	non-AMA	12	increased nuchal translucency; hypoplastic nasal bone	positive	high risk	21-T	terminated
NO.8	non-AMA	26	• persistent left superior vena cava; coronary sinus dilatation	positive	high risk	21-T	terminated
NO.9	non-AMA	24	perimembranous ventricular septal defect; micromandible; low-set ears; unilateral complete cleft palate (III)	positive	high risk	18-T	terminated
NO.10	non-AMA	28	increased nuchal fold; Dandy-Walker malformation; double outlet right ventricle	positive	high risk	18-T	terminated
NO.11	non-AMA	33	left ventricular hyperechoic plaques; polycystic renal dysplasia; semilobarholoprosencephaly; cyclopia; beak nose; intrauterine growth restriction	negative	high risk	13-T	terminated

The incidence of chromosomal abnormalities in AMA group was much higher than in non-AMA group (*χ*^2^ = 13.79, df = 1, *P* < 0.001, OR = 0.46, 95% CI: 0.31–0.69). While there was no difference in ARSA incidence between the AMA and non-AMA groups (*χ*^2^ = 1.39, df = 1, *P* = 0.24, OR = 1.36, 95% CI: 0.856–2.14), and in the isolated and combined ARSA group (*χ*^2^ = 0.17, df = 1, *P* = 0.68, OR = 1.18, 95% CI: 0.66–2.12 and *χ*^2^ = 1.50, df = 1, *P* = 0.22, OR = 1.7, 95% CI: 0.82–3.65). The risk of chromosomal abnormalities significantly increased with ARSA detection (*χ*^2^ = 182.77, df = 1, *P* < 0.001, OR = 21.70, 95% CI: 11.44–41.14). Among AMA and non-AMA ARSA-positive cases, the incidence increased to 20.00% (*χ*^2^ = 69.11, df = 1, *P* < 0.001, OR = 21.90, 95% CI: 7.74–61.96) and 10.00% (*χ*^2^ = 104.35, df = 1, *P* < 0.001, OR = 20.50, 95% CI: 9.008–46.66), respectively. The OR for AMA and non-AMA was homogeneous (*χ*^2^ = 0.01, *P* = 0.92). Therefore, age was not a confounding factor for the association between ARSA and aneuploidy. The Mantel-Haenszel Common OR estimate was 21.06, 95% CI: 11.05–40.15, *P* < 0.001. Similarly, with additional ultrasonic findings, chromosomal abnormalities risk increased simultaneously in the AMA and non-AMA groups (Fisher’s exact test, all *P* < 0.001).

The likelihood ratio and predictive value of ARSA for chromosomal abnormalities are displayed in Table 3. In the AMA group, the likelihood ratio of combined ARSA for chromosomal abnormalities was 246.00, higher than that of the entire cohort and non-AMA group (69.76 and 97.82, respectively, all *P* < 0.001).

**Table 3:** The independently predictive value of ARSA or combined ARSA for chromosomal abnormalities

Group	Index	Sensitivity (95% CI)	Specificity (95% CI)	Positive predictive value (95% CI)	Negative predictive value	Likelihood ratio
Entirecohort	ARSA	11.76% (6.23–19.65) %	99.39% (99.24–99.51) %	12.63% (6.70–21.03) %	99.34% (99.19–99.47) %	19.26
	Combined ARSA	10.78% (5.51–18.48) %	99.85% (99.76–99.90) %	34.38% (18.57–53.19) %	99.33% (99.18–99.46) %	69.76
AMA	ARSA	13.51% (4.54–28.77) %	99.29% (98.91–99.57) %	20.00% (6.83–40.70) %	98.87% (98.41–99.23) %	19.07
	Combined ARSA	10.81% (3.03–25.42) %	99.96% (99.90–99.98) %	40.00% (12.16–73.76) %	99.76% (99.66–99.83) %	246.00
non-AMA	ARSA	10.77% (4.44–20.94) %	99.41% (99.25–99.55) %	10.00% (4.12–19.52) %	99.46% (99.30–99.59) %	18.40
	Combined ARSA	10.77% (4.44–20.94) %	99.89% (99.82–99.94) %	31.82% (13.86–54.87) %	99.58% (99.45–99.68) %	97.82

*ARSA* Aberrant right subclavian artery, *AMA* Advanced maternal age, *non-AMA* appropriate maternal age, *OR* Odds ratio, *CI* Confidence intervals

## Discussion

In this retrospective study, 13,690 singleton pregnancies were evaluated. We found that the ARSA incidence was 0.87% in Chinese AMA and 0.65% in non-AMA women. The incidence of chromosomal abnormalities was much higher in the AMA group than that in the non-AMA group. In the ARSA-positive group, the incidence was 20% in the AMA group and 10% in the non-AMA group. However, age did not affect the incidence of ARSA, nor did it affect aneuploidy in ARSA-positive patients. The likelihood ratio of combined ARSA for chromosomal abnormalities was high in the AMA group. Among aneuploidy groups, the most-commonly detected was Down syndrome in both groups. A child with chimeric Turner syndrome with an isolated ARSA was born to an AMA woman.

In most studies, the prevalence of a prenatal ARSA was studied in the entire population with an incidence ranging from 0.4 to 1.5% [14, 15]. Concordant with the previous studies, we found the incidence of ARSA in the entire cohort was 0.69%. Furthermore, we confirmed there was no difference in ARSA incidence in the AMA and non-AMA groups. Regarding the timing of prenatal ultrasonic diagnosis of ARSA, Pico et al. reported that the mean gestational age for ARSA detecting was 19 weeks + 5 days, ranging from 11 weeks + 5 days to 34 weeks, SD = 4 days [6]. In another study concerning the predictive value of ARSA for Down syndrome, authors checked pregnant women at 16 weeks of gestation [12]. In our experience, ARSA can be detected as early as 12 weeks + 4 days gestational age. However, due to the high omission diagnostic rate during the first trimester and the early mid-trimester, and to reduce the bias caused by the difference in technical level between examiners and ensure accuracy, we set the threshold 16 weeks of gestation for this study.

The association between ARSA fetuses and chromosomal abnormalities such as Down syndrome was described previously [12, 16, 17]. Paladini et al. stated that ARSA should be considered among the three most potent ultrasound indicators of Down syndrome in the second trimester; the appearance resembles nasal bone abnormalities and increased nuchal folds [15]. As we found, ARSA increased the risk of chromosomal abnormalities, especially combined ARSA.

Regarding isolated ARSA, a weak association between isolated ARSA and chromosomal abnormalities were reported by other scholars [14]. Pico et al. suggested that these fetuses with isolated ARSAs required a comprehensive evaluation instead of an invasive karyotype analysis because isolated ARSA is rarely associated with chromosomal abnormalities [6]. In our study, only one AMA with an isolated ARSA fetus had a chromosomal abnormality, a chimera (45X / 46XX). Turner syndrome is closely associated with cardiovascular malformations with a frequency of 23 to 45%. Lee et al. reported that ARSA was a common significant vessel abnormality in the Turner syndrome patients (3 / 20 patients, 15%) [18]. Due to our small sample size, we did not have enough evidence to show that ARSA was related to chimerism (45X / 46XX). The effect of NIPT in AMA pregnancy was discussed in a multicenter retrospective study [19]. The authors found a positive predictive value of 41.30% for sex chromosome aneuploidy in AMA pregnancy. Most mothers in our study did not undergo invasive karyotype analysis; we cannot exclude chimera from other fetuses with NIPT. Therefore, this isolated ARSA case must be considered a random event, and we excluded it from statistical analysis. Nevertheless, vigilance is required when an isolated ARSA is found. An active prenatal counseling and a comprehensive prenatal assessment would be conducive to further managing.

We found age did not affect the incidence of ARSA, nor did it affect the incidence of aneuploidy in ARSA positive patients. The Mantel-Haenszel Common OR estimated was 21.06. After removing confounding factors, in ARSA-positive patients, aneuploidy’s risk was almost 20 times higher than in ARSA-negative patients. This study’s results are supported by Chen et al. [5]; there is no need for AMA women carrying fetuses with ARSA to undergo invasive prenatal diagnosis directly; however, if AMA women have a fetus with combined ARSA, the risk of aneuploidy is significantly higher.

Concerning combined ARSA, in virtue of prenatal detection of ARSA with other ultrasound signs, the risk for trisomy 21 increased by a factor of 45, according to Fehmi et al. [12]. As in our study, the likelihood ratios of combined ARSA for chromosomal abnormalities in the entire population, AMA and non-AMA groups were 69.76, 246.00, and 97.82, respectively. Svirsky et al. supported our results; they claimed ARSA with additional ultrasound findings constituted a strong predictor for aneuploidy [16].

There were some limitations in our study. First, several fetuses in the non-AMA group did not undergo invasive karyotype analysis. The significant aneuploidy abnormalities and karyotype abnormalities were excluded by the negative results of noninvasive DNA tests, detailed prenatal examinations, and neonatal follow-up. In clinical practice, many parents are reluctant to undergo invasive karyotype analysis considering the possible risks of invasive procedures. In theory, an isolated ARSA is not sufficient to indicate karyotype analysis [6]. Ranzini et al. reported that all fetuses with ARSA and genetic anomalies had additional ultrasound findings [14]. In similar studies, the authors included fetuses with consistently classified fetuses with negative prenatal screening and postpartum follow-up as normal karyotypes [6, 7]. Therefore, we believe that the method adopted in our study is acceptable. Second, chromosomal microarray analysis was not analyzed in the current study.

Nevertheless, it is worth noting that several deformities might be neglected without chromosomal microarray analysis, according to Maya et al. [17]. Finally, the incidence of ARSA in pregnancies over the age of 40 and its predictive value for chromosome abnormality was not evaluated individually in our current study. This limitation may be significant in this group. We look forward to advancing the discussion of these issues in future studies.

## Conclusion

The incidence of ARSA in Chinese AMA women resembled that of non-AMA women. ARSA increased the risk of chromosomal abnormalities in both age groups. There was a high prevalence of chromosomal abnormalities in AMA fetuses. When combined ARSA was found in AMA ones, it conferred a high likelihood of chromosomal abnormalities. The incidence of ARSA in women over the age of 40 and its predictive value for chromosome abnormality merits further investigation.

## Data Availability

The datasets used and analyzed during the current study are available from the corresponding author on reasonable request.

## Abbreviations

ARSA: Aberrant right subclavian artery
AMA: Advanced maternal age; non-AMA, appropriate maternal age
OR: Odds ratio
CI: Confidence intervals
NIPT: Noninvasive prenatal testing

## References

[1] Lean SC, Derricott H, Jones RL, Heazell AEP (2017). Advanced maternal age and adverse pregnancy outcomes: a systematic review and meta-analysis. PLoS One.

[2] Zeng Y, Hesketh T (2016). The effects of China's universal two-child policy. Lancet..

[3] Laopaiboon M, Lumbiganon P, Intarut N, Mori R, Ganchimeg T, Vogel JP, Souza JP, Gülmezoglu AM (2014). WHO Multicountry Survey on Maternal Newborn Health Research Network. Advanced maternal age and pregnancy outcomes: a multicountry assessment. BJOG.

[4] Zhu Y, Lu S, Bian X, Wang H, Zhu B, Wang H, Xu Z, Xu L, Yan W, Zeng Y, Chen Z, Tang S, Shen G, Miao Z (2016). A multicenter study of fetal chromosomal abnormalities in Chinese women of advanced maternal age. Taiwan J Obstet Gynecol.

[5] Chen Y, Zheng XL, Wu SW, Zhang WY (2017). Clinic characteristics of women with advanced maternal age and perinatal outcomes. Zhonghua Fu Chan Ke Za Zhi.

[6] Pico H, Mancini J, Lafouge A, Bault JP, Gorincour G, Quarello E (2016). Prenatal associated features in fetuses diagnosed with an aberrant right subclavian artery. Fetal Diagn Ther.

[7] Gursoy Erzincan S, Karamustafaoglu Balci B, Tokgoz C, Kalelioglu IH (2017). Incidence of an aberrant right subclavian artery on second - trimester sonography in an unselected population. J Ultrasound Med.

[8] Willruth AM, Dwinger N, Ritgen J, Stressig R, Geipel A, Gembruch U, Berg C (2012). Fetal aberrant right subclavian artery (ARSA) - a potential new soft marker in the genetic scan?. Ultraschall Med.

[9] Chaoui R, Thiel G, Heling KS (2005). Prevalence of an aberrant right subclavian artery (ARSA) in normal fetuses: a new soft marker for trisomy 21 risk assessment. Ultrasound Obstet Gynecol.

[10] Scala C, Leone Roberti Maggiore U, Candiani M, Venturini PL, Ferrero S, Greco T, Cavoretto P (2015). Aberrant right subclavian artery in fetuses with Down syndrome: asystematic review and meta-analysis. Ultrasound Obstet Gynecol.

[11] Sagi-Dain L, Singer A, Josefsberg S, Peleg A, Lev D, Samra NN, Bar-Shira A, Zeligson S, Maya I, Ben-Shachar S (2019). Microarray analysis has no additional value in fetal aberrant right subclavian artery: description of 268 pregnancies and systematic literature review. Ultrasound Obstet Gynecol.

[12] Fehmi Yazıcıoğlu H, Sevket O, Akın H, Aygün M, Özyurt ON, Karahasanoğlu A (2013). Aberrant right subclavian artery in Down syndrome fetuses. Prenat Diagn.

[13] De León-Luis J, Gámez F, Bravo C, Tenías JM, Arias Á, Pérez R, Maroto E, Aguarón Á, Ortiz-Quintana L (2014). Second-trimester fetal aberrant right subclavian artery: original study, systematic review and meta-analysis of performance in detection of Down syndrome. Ultrasound Obstet Gynecol.

[14] Ranzini AC, Hyman F, Jamaer E, van Mieghem T (2017). Aberrant right subclavian artery: correlation between fetal and neonatal abnormalities and abnormal genetic screening or testing. J Ultrasound Med.

[15] Paladini D, Sglavo G, Pastore G, Masucci A, D'Armiento MR, Nappi C (2012). Aberrantright subclavian artery: incidence and correlation with other markers of Down syndrome in second - trimester fetuses. Ultrasound Obstet Gynecol.

[16] Svirsky R, Reches A, Brabbing-Goldstein D, Bar-Shira A, Yaron Y (2017). Association of aberrant right subclavian artery with abnormal karyotype and microarray results. Prenat Diagn.

[17] Maya I, Kahana S, Yeshaya J, Tenne T, Yacobson S, Agmon-Fishman I (2017). Cohen-VigL, Levi a, Reinstein E, Basel-Vanagaite L, Sharony R. chromosomal microarray analysis in fetuses with aberrant right subclavian artery. Ultrasound Obstet Gynecol.

[18] Lee SH, Jung JM, Song MS, Sj C, Chung WY (2013). Evaluation of cardiovascular anomalies in patients with asymptomatic turner syndrome using multidetector computed tomography. J Korean Med Sci.

[19] Yu B, Li H, Chen YP, Zhang B, Xue Y, He Q, Zhou Q, Cai Z, Wang T (2019). Clinical evaluation of NIPS for women at advanced maternal age: a multicenter retrospective study. J Matern Fetal Neonatal Med.

